# TIME-COURSE FOR ACQUIRING TRANSFER INDEPENDENCE IN PATIENTS WITH SUBACUTE STROKE: A PROSPECTIVE COHORT STUDY

**DOI:** 10.2340/jrm.v56.40055

**Published:** 2024-10-09

**Authors:** Shin KITAMURA, Yohei OTAKA, Shintaro UEHARA, Yudai MURAYAMA, Kazuki USHIZAWA, Yuya NARITA, Naho NAKATSUKASA, Daisuke MATSUURA, Rieko OSU, Kunitsugu KONDO, Sachiko SAKATA

**Affiliations:** 1 Department of Rehabilitation Medicine, Tokyo Bay Rehabilitation Hospital, Chiba; 2 Faculty of Rehabilitation, School of Health Sciences, Fujita Health University, Aichi; 3 Department of Rehabilitation Medicine, School of Medicine, Fujita Health University, Aichi; 4 Faculty of Rehabilitation, School of Health Sciences, Chiba Prefectural University of Health Sciences, Chiba; 5 Faculty of Human Sciences, Waseda University, Saitama, Japan

**Keywords:** activities of daily living, cerebrovascular disorders, cluster analysis, task performance, wheelchair

## Abstract

**Objective:**

To clarify the time-course of longitudinal changes in the independence level of subtasks composing bed–wheelchair transfer among patients with stroke.

**Design:**

Single-institution prospective cohort study.

**Patients:**

A total of 137 consecutive post-stroke patients using wheelchair on admission to the subacute rehabilitation wards.

**Methods:**

The independence degree in each of the 25 transfer-related subtasks was assessed using the Bed–Wheelchair Transfer Tasks Assessment Form on a three-level scale every two weeks, from admission to the endpoint (either discharge or when achieving independent transfer). Patients were classified based on admission and endpoint assessment form scores using two-step cluster analysis.

**Results:**

Patients were classified into three clusters. The first cluster included 50 patients who exhibited a greater independence level in all subtasks on admission (52.0–100% of patients performed each subtask independently) and at the endpoint (64.0–100%). The second included 30 patients who showed less independence on admission (0–27.8%) but achieved greater independence levels at the endpoint (44.4–97.2%). The third included 51 patients whose independence level remained low in many subtasks from admission (0–5.8%) until the endpoint (0–29.4%).

**Conclusion:**

The independence level and its changing process during transfer were categorized into three time-courses, each requiring different intervention strategies.

## LAY ABSTRACT

Transferring, which consists of subtasks, is essential for patients with stroke to expand their activities of daily living. Understanding potential processes towards independence in transfer-related subtasks and identifying specific subtasks that tend to remain dependent can help identify subtasks to be practised with priority and to develop effective strategies for promoting independence. We longitudinally examined the independence level and its change process in individual subtasks comprising “bed–wheelchair transferring” in 137 patients with stroke admitted to rehabilitation wards. Patients were classified into three patterns: those with greater independence level in all subtasks on admission and at discharge; low independence level on admission but achieved greater independence levels at discharge; and low independence level in many subtasks from admission to discharge. These findings can be used to develop tailored rehabilitation strategies for each patient’s time-course of transfer independence in the early stages of hospitalization.

For patients with stroke, regaining activities of daily living (ADL) is a major rehabilitation goal. Early mobilization (i.e., leaving the bed) is crucial to regain ADL after stroke (1). To leave the bed, many patients with stroke need a wheelchair during their hospital stay due to walking difficulties (2). Therefore, acquiring the ability to transfer between a wheelchair and a bed at an early stage of hospitalization is an important step towards regaining ADL.

To date, the effectiveness of various types of interventions on overall ADLs in stroke survivors has been demonstrated, including occupational therapy (3), caregiver-mediated exercise (4), dual-task proprioceptive training (5, 6), and interventions using the latest technology, such as virtual reality (7, 8). In addition, factors affecting the acquisition of ADLs have been identified as internal factors, such as patient demographics (e.g., age [9–12] and sex [11, 13–15]), and clinical characteristics (e.g., motor [13, 16] and cognitive functions [14, 16]), as well as external factors such as infections (17, 18) and the organization of services in rehabilitation (19). Although related evidence on the acquisition of overall ADL in stroke survivors is accumulating, few reports have provided specific strategies for acquiring transfer skills (20), and these strategies are not well established. The bed-wheelchair transfer is a serial task comprising multiple subtasks (21). For example, these include manoeuvring the wheelchair and approaching the bed, braking, taking the foot off the footrest and placing it on the ground, and standing up and changing direction (22). We identified 25 transfer-related subtasks and developed the Bed–Wheelchair Transfer Tasks Assessment Form (BTAF), a tool to assess the independence level for each subtask (22). Each subtask is independent to some degree (22) and varies in difficulty to complete (23). Therefore, the overall transfer’s independence is constrained by the extent of acquired subtasks. That is, the process towards acquiring independence for the overall transfer depends on the time-course of acquiring independence for each subtask. For example, a patient with a low independence level for the overall transfer upon admission may have either no change or an increase in independence level for all subtasks, or they may increase their independence level for specific subtasks during hospitalization. Subtask independence attainment is influenced by the severity of post-stroke symptoms and demographic characteristics.

Patients with stroke exhibit diverse clinical characteristics influenced by the location or volume of the brain damage (24–26) and demographic characteristics, such as age and sex. Therefore, the processes towards independence may also show different time-courses due to the interaction between the difficulty of each element of the transfer skill (23) and individual clinical and demographic characteristics. If so, tailored rehabilitation strategies are essential, especially for groups of patients with different time-courses in gaining transfer skills independence. Patients anticipating difficulty in acquiring the required independence level may prioritize simpler subtasks and utilize compensatory measures, such as assistive devices, for difficult-to-acquire subtasks. Understanding potential processes toward independence in transfer-related subtasks and identifying specific subtasks that tend to remain dependent can help patients prioritize which subtasks to practise and develop effective strategies for promoting independence.

This prospective cohort study aimed to clarify the differences in time-course and associated factors towards independence in bed-wheelchair transfer-related subtasks among patients with stroke. We classified patients into subtypes based on the time-course of changes in the independence level of subtasks during hospitalization and investigated the characteristics of their time-course and patients’ demographics within each subtype.

## METHODS

### Study design and setting

This single-centre prospective cohort study adhered to the Strengthening the Reporting of Observational Studies in Epidemiology Statement guidelines (Table SI) (27). The study was conducted in the Kaifukuki Rehabilitation Ward of Tokyo Bay Rehabilitation Hospital, a 160-bed facility in Japan. This ward provides subacute intensive rehabilitation covered by Japan’s medical insurance system, admitting patients with stroke within two months of symptom onset for a maximum stay of six months (28). The sample size was determined based on the planned study period (April 2016 and March 2017). The Ethics Committee of Tokyo Bay Rehabilitation Hospital approved the study protocol (approval no. 135), and all participants provided written informed consent.

### Participants

Patients hospitalized after stroke were consecutively recruited between April 2016 and March 2017. Inclusion criteria comprised first ischaemic or haemorrhagic stroke, hemiparesis with apparent unilateral motor paresis on the motor items in the Stroke Impairment Assessment Set (SIAS) upon admission, use of wheelchair upon admission for daily mobility, and provision of consent via a legal representative.

### Procedure

The patient’s occupational therapist assessed the independence level of bed–wheelchair transfer by observing the actual performance in the hospital room using the BTAF (22). BTAF is a tool developed specifically to assess the bed-to-wheelchair transfer of patients with paretic stroke (Fig. S1). The BTAF classifies a series of transfer tasks into 25 subtasks (Table I). Each subtask independence level is assessed on a three-point scale: 3, independent (participant can complete the task by themselves without any assistance from a therapist); 2, requiring supervision or verbal assistance (participant can complete a task under supervision or verbal assistance from a therapist); 1, requiring physical assistance (participant needs physical assistance from a therapist, such as locking the wheelchair brakes and manoeuvring the wheelchair to the appropriate position) to complete the task; and N, not applicable (participant does not need to perform the task: for example, the task of “put the foot on the footrest” can be applied only to those using wheelchairs with footrests). The mean subtask score was calculated to indicate the overall BTAF score for each patient. Subtasks marked as “N, not applicable” were excluded, and the mean score was calculated from the remaining subtasks (i.e., those judged as “1”, “2”, or “3”). For example, if 2 of the 25 subtasks were marked as “N”, the mean score was calculated based on the other 23 subtasks. This assessment was demonstrated to have good reliability and validity (22).

**Table I:** Subtasks comprising the Bed–Wheelchair Transfer Tasks assessment form

Transferring task	Subtask
Bed-to-wheelchair	Press the nurse call button (bed to WC)
	Take off the comforter
	Manipulate the handrail for the bed
	Roll over
	Get up
	Keep sitting at the bedside (bed to WC)
	Wear shoes/brace
	Ready the wheelchair for transfer (the position of the wheelchair, brakes, and footrests)
	Stand up from the bed
	Turn while standing (bed to WC)
	Sit on the wheelchair seat
	Put a foot on the footrest
	Unlock the wheelchair brakes
	Manoeuvre the wheelchair
Wheelchair-to-bed	Press the nurse call button (WC to bed)
	Manoeuvre the wheelchair towards the appropriate place for transfer to the bed
	Lock the wheelchair brakes
	Take the foot off the footrest and place it on the ground
	Stand up from sitting in the wheelchair
	Turn while standing (WC to bed)
	Sit on the bed
	Keep sitting at the bedside (WC to bed)
	Take off shoes/brace
	Lie down on the bed
	Put on the comforter

WC: wheelchair. The items are listed in order of their performance.

During the assessment, patients were instructed to perform bed-to-wheelchair transfer and wheelchair-to-bed transfers daily for three days. The lowest score of the three was used as the one representing the independence level of the subtask (if a patient’s performance for one subtask was scored as “3”, “3”, and “2”,”2” was adopted).

The BTAF assessment was conducted by well-trained occupational therapists with daily clinical practice in the hospital. Patients were assessed for their performance in a hospital room setting where they routinely perform transfers. In this hospital, patients typically used wheelchairs with flip-up arm supports and removable foot supports, and L-shaped bed rails that opened 90 degrees. The room environment was adjusted by nurses and therapists to ensure the highest level of patient independence. For example, the bed height was set according to the individual patient’s ability to easily stand up and maintain a sitting position. Assessments were conducted every two weeks from admission until reaching one of the following endpoints: (*i*) Independence, when all subtasks were rated as “3, independent” or “N, not applicable,” or when the patient received permission from the medical team to perform the transfer alone even if some subtasks were rated “2, requiring supervision or verbal assistance” or “1, requiring physical assistance” (i.e., if a patient was judged to be independent based on an assessment by the medical team separately from the study, we defined the previous two-week assessment time as the endpoint, even if not all subtasks were rated as “3”); (*ii*) Mobility change, when patients no longer used a wheelchair because they began to ambulate; (*iii*) Discharge, when patients were discharged from the hospital regardless of the independence level.

Regarding patients’ clinical characteristics on admission, the SIAS (29, 30), Mini-Mental State Examination-Japanese (MMSE-J) (31, 32), and Functional Independence Measure (FIM) (33, 34) were obtained on admission to the wards. These assessments have been verified for reliability and validity in patients post-stroke (29, 30, 35–38).

### Data analysis

The dataset of a single patient consisted of the independence ratings of the 25 BTAF subtasks for the number of times the assessment (mean 5.5 times) was completed. We adopted a two-step cluster analysis to classify patients into subgroups based on the time-course of independence in bed–wheelchair transfer subtasks. In the cluster analysis, the assessment results at two time points were used: on admission and at the endpoint. These were combined into categorical variables (e.g., “1–2” for a patient rated “1” on admission and “2” at the endpoint), and 25 categorical variables per patient were evaluated. In the two-step cluster analysis, several pre-clusters were first created based on the distance measure, and then smaller clusters were combined stepwise in the next step through hierarchical cluster analysis. The two-step cluster analysis was employed because it can include categorical variables, and the number of best-fitting clusters can be automatically determined. Log-likelihood was used as the distance measure, and Schwarz’s Bayesian criterion was used to determine the cluster number. Subsequently, clustering quality was evaluated using silhouette coefficients. The coefficients range from –1 to 1, with –1 to 0.2 indicating poor; 0.2 to 0.5, fair; and ≥ 0.5, good (39).

To characterize clusters, we calculated the percentage of patients corresponding to each of the BTAF scales (3, 2, 1, N) for each subtask on admission and at the endpoint for each cluster. Demographic data were compared among the clusters to identify patient characteristics, using the χ^2^ test for nominal data, one-way analysis of variance for proportional scale data, and the Kruskal–Wallis test for ordinal data.

Cluster analysis and the subsequent analyses were performed using SPSS version 28 (IBM Corp, Armonk, NY, USA). Any *p*-values < 0.05 were considered statistically significant.

## RESULTS

**Fig. 1:**
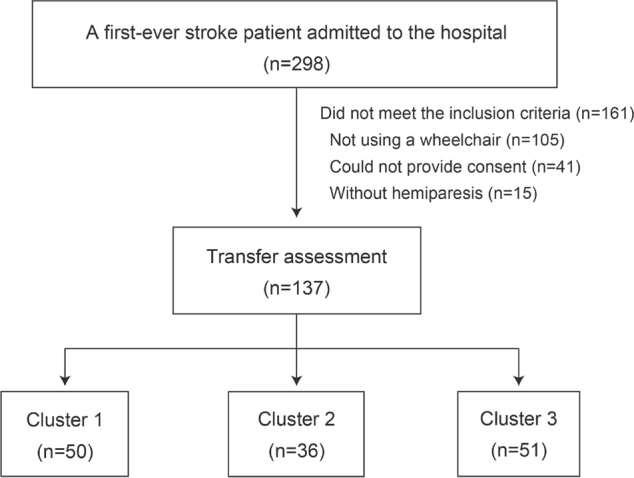
Flowchart of sampling and clustering. Patients with stroke (*n* = 137) were classified into three clusters using cluster analysis.

Among the 298 patients admitted with their first stroke during the study period, 137 consecutive patients who met the criteria were included (Fig. 1). The patient characteristics are presented in Table II.

**Table II:** Demographic and clinical characteristics of the participants

Factor	Cluster 1 (*n* = 50)	Cluster 2 (*n* = 36)	Cluster 3 (*n* = 51)	Comparison of all clusters *p*-value
Age, years, mean (SD)	65.2 (12.2)	66.9 (13.8)	76.5 (9.2)	< 0.001
Sex, *n* (% in each cluster)				
Male	36 (72.0)	21 (58.3)	22 (43.1)	0.013
Female	14 (28.0)	15(41.7)	29 (56.9)	
Type of stroke, *n* (% in each cluster)				
Haemorrhage	17 (34.0)	18 (50.0)	21 (41.2)	0.200
Infarction	33 (66.0)	16 (44.4)	29 (56.9)	
Subarachnoid haemorrhage	0 (0.0)	2 (5.6)	1 (2.0)	
Duration after stroke onset, days, mean (SD)	32.1 (13.1)	38.9 (15.8)	41.6 (14.0)	0.004
Paretic side, *n* (% in each cluster)				
Right	25 (50.0)	24 (66.7)	25 (49.0)	0.206
Left	25 (50.0)	12 (33.3)	26 (51.0)	
Duration of assessment, weeks, mean (SD)	4.6 (5.4)	9.3 (5.5)	13.3 (6.8)	< 0.001
Reason for ending the assessment, *n* (% in each cluster)				
Independence in transferring	34 (68.0)	14 (38.9)	0 (0.0)	< 0.001
Independence in transferring with changing mobility from wheelchair to walking	13 (26.0)	12 (33.3)	4 (7.8)	
Discharge (not acquiring independence in transferring)	3 (6.0)	10 (27.8)	47 (92.2)	
MMSE, median (IQR)	28 (4)*	21 (15)^†^	15 (11)^‡^	< 0.001
FIM, median (IQR)				
Motor score	50.5 (12.5)	26.5 (14.5)	18 (8)	< 0.001
Cognitive score	28.5 (9)	20 (9.5)	14 (8)	< 0.001
Total score	78 (20)	45.5 (21.5)	33 (13)	< 0.001
SIAS, median (IQR)				
Knee–mouth	4 (2)	1 (4)	0 (2)	< 0.001
Finger function	3.5 (3)	1 (4)	0 (1)	< 0.001
Hip flexion	4 (2)	2 (4)	0 (2)	< 0.001
Knee extension	4 (1)	2 (3.5)	0 (2)	< 0.001
Foot-pat	3 (2)	2 (4)	0 (2)	< 0.001
Visuospatial	3 (0)^§^	3 (0)^||^	3 (2)^¶^	0.143
Speech	3 (1)	2 (2)	2 (2)^#^	0.030

IQR: interquartile range; SIAS: Stroke Impairment Assessment Set; MMSE: Mini-Mental State Examination; FIM: Functional Independence Measure, missing data:

* = 4,

† = 12,

‡ = 22,

§ = 2,

|| = 3,

¶ = 5, and

# = 1.

**Fig. 2:**
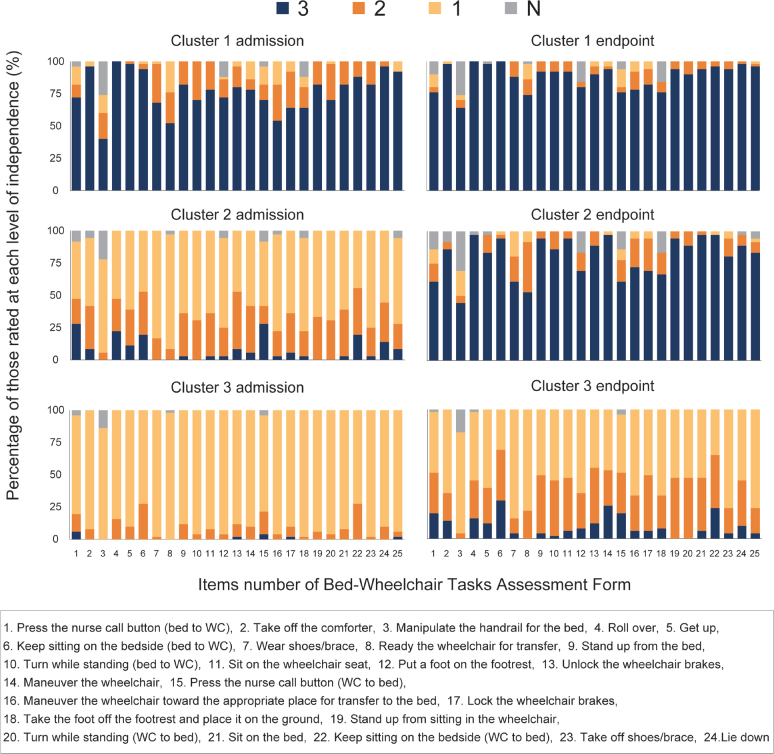
Independence level of each subtask on admission and at the endpoint in each cluster. The items are listed in order of their performance. Each subtask is assessed as “3, independent”, “2, requires supervision or verbal assistance”, “1, requires assistance”, and “N, not applicable”.

**Fig. 3:**
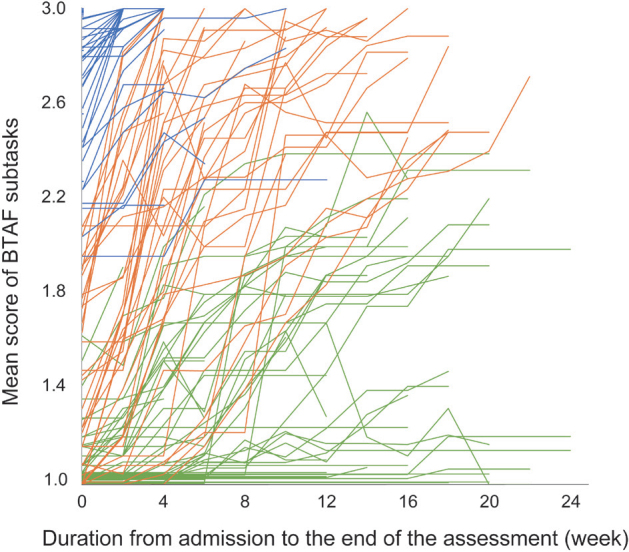
The mean score of BTAF subtasks at each assessment point for individual patients. Each line shows the BTAF scores of individual patients, with blue for those in Cluster 1, orange for Cluster 2, and green for Cluster 3. The mean score of BTAF subtasks was calculated by dividing the total score by the number of items excluding those judged “N, not applicable”. BTAF: Bed-Wheelchair Transfer Tasks assessment form.

The patients were classified into three clusters using the two-step cluster analysis: 50 patients (36.5%) were assigned to Cluster 1, 36 (26.3%) to Cluster 2, and 51 (37.2%) to Cluster 3. Silhouette coefficients were 0.4, indicating that the clustering quality was “fair”. Fig. 2 shows the results of BTAF assessments for each subtask on admission and the endpoint in each cluster. Cluster 1 showed the highest percentage of “3” ratings for all items on admission (52.0–100%, mean 77.0%) and at the endpoint (64.0–100%, mean 88.5%). Cluster 2 displayed the highest percentage of “1” in all items on admission (44.4–88.8%, mean 63.0%) and the highest percentage of “3” at the endpoint (44.4–97.2%, mean 80.4%). Cluster 3 showed the highest percentage of “1” on admission (72.5–98.0%, mean 90.0%) and at the endpoint (31.3–84.3%, mean 57.8%) for most items (23/25, 92.0%). The time-course of mean BTAF subtask scores for individual patients is illustrated in Fig. 3. Those in Cluster 1 had relatively high scores on admission and showed rapid improvement. Those in Cluster 2 had low scores on admission but significantly improved by the endpoint, while Cluster 3 patients, despite gradual improvement, maintained relatively low scores even at the endpoint.

Table II presents the patient characteristics and results of statistical comparisons among the clusters. Statistically significant differences were observed in age (*F* [2,134] = 13.00, *p* < 0.001), sex (*c*^2^ [2] = 8.62, *p* = 0.013), duration after stroke onset (*F* [2, 134] = 5.78, *p* = 0.004), duration of transfer assessment (*F* [2, 134] = 14.57, *p* < 0.001), MMSE (*H* [2] = 41.09, *p* < 0.001), FIM (motor: *H* [2] = 80.26, *p* < 0.001, cognitive: *H* [2] = 50.27, *p* < 0.001, and total scores: *H* [2] = 80.47, *p* < 0.001), and SIAS (upper extremity motor: *H* [2] = 24.96–34.25, *p* < 0.001, lower extremity motor: *H* [2] = 25.87–39.34, *p <* 0.001, and speech items: *H* [2] = 7.02, *p* = 0.030). A trend towards older age, longer post-onset and transfer assessment periods, fewer males, and lower cognitive and motor functions were evident from Cluster 1 to Cluster 3.

Regarding the reasons for ending the assessment (endpoint status), “independence of transferring” was the main reason for patients in Cluster 1 (68.0%), decreasing to 38.9% in Cluster 2 and 0% in Cluster 3. Simultaneously, the percentage of patients who ended the assessment due to “discharge” increased from Cluster 1 (6.0%), to Cluster 2 (27.8%), to Cluster 3 (92.2%, *c*^2^ [4] = 86.52 *p* < 0.001). Notably, “independence of transferring with changing mobility from wheelchair to walking” was the most common reason in Cluster 2 (33.3%).

Regarding the independence level of each subtask for each cluster (Fig. 2), the subtasks with the lowest percentage of “3” at the endpoint were those related to preparation for transfer, such as preparing bed rails, nurse calls, and wheelchairs in Cluster 1 and Cluster 2. In particular, Cluster 2 contained at least one-third of dependent patients (i.e., who were rated as “2” or “3”) in subtasks such as “Press the nurse call button (bed to wheelchair)”, “Manipulate the bed handrail”, “Wear shoes/brace”, “Ready the wheelchair for transfer”, “Press the nurse call button (wheelchair to bed)”, and “Take the foot off the footrest”. Conversely, more than 90% of patients were rated “3” in the subtasks of “Roll over”, “Keep sitting on the bedside (bed to wheelchair)”, “Stand up from the bed”, “Sit on the wheelchair seat”, “Manoeuvre the wheelchair”, “Stand up from sitting in the wheelchair”, “Sit on the bed”, and “Keep sitting on the bedside (wheelchair to bed)”. In Cluster 3, only two subtasks, “Keep sitting on the bedside (bed to wheelchair)” and “Keep sitting on the bedside (wheelchair to bed)”, had the highest percentage of “2” at the endpoint, while the other subtasks had the highest percentage of “1” both on admission and at the endpoint. Especially in subtasks including “Manipulate the handrail for the bed”, “Wear shoes/brace”, “Ready the wheelchair for transfer”, “Manoeuvre the wheelchair to the bed”, “Take the foot off the footrest”, and “Take off shoes/brace”, at least two-thirds of the patients remained classified as “1” at the endpoint (indicating a need for assistance). In contrast, in the subtasks “Press the nurse call button (bed to wheelchair)”, “Keep sitting at the bedside (bed to wheelchair)”, “Unlock the wheelchair brakes”, “Manoeuvre the wheelchair”, “Press the nurse call button (wheelchair to bed)”, and “Keep sitting at the bedside (wheelchair to bed)”, more than half the patients were rated as “3” or “2” in endpoint, and relatively few patients remained rated as “1”.

## DISCUSSION

To understand the time-course toward independence in transfer-related subtasks on admission, we classified patients with subacute stroke based on the time-course in the level of independence of subtasks comprising bed–wheelchair transfer into three clusters: Cluster 1, patients who showed near independence in many subtasks on admission and then became independent early during hospitalization; Cluster 2, patients who required assistance on admission but became independent during hospitalization; Cluster 3, patients requiring assistance on admission and remaining dependent at discharge.

The study identified three types of patients after stroke based on their time-course of independence in the subtasks comprising transfer. These findings corroborate a previous study on the time-course of FIM composite scores in patients with stroke admitted to a subacute rehabilitation unit (40). The previous study identified three types of patients: those with high scores on admission and at discharge, those with intermediate scores on admission that improved significantly by their discharge, and those with low scores throughout their hospital stay (40). Importantly, the existence of patients who required assistance on admission but whose level of independence improved significantly during hospitalization has been widely observed in many studies that examined the time-course of independence levels in ADL after stroke (41–44).

Comparing patient characteristics across clusters, we observed that younger age, shorter post-onset time, and duration between the initial and endpoint assessments were associated with higher independence percentages, progressing from Cluster 3 to 1. Additionally, male patients with higher cognitive and motor functions were more prevalent in clusters with greater independence. Previous studies have consistently reported a similar association, linking younger age (9–12), male sex (11, 13–15), shorter post-onset time (11, 45), and mild motor (13, 16), cognitive (14, 16), and language (46) dysfunctions to improved ADL in patients with stroke. The present findings indicate that many patient characteristics showed gradual changes from Cluster 1 to Cluster 3, suggesting that multiple characteristics, such as the severity of poststroke sequelae and other factors associated with the level of ADL independence (e.g., age and sex ) (9–16, 45), rather than a single characteristic, contribute to classifying the patient groups.

Patients in Cluster 1, characterized by high level of independence in many subtasks on admission and mild motor and cognitive impairment, are likely to achieve independence in transfer using a wheelchair early during hospitalization, facilitating early mobilization, crucial for ADL functional independence (1). However, it should be noted that, even within this group, some patients are not initially independent in performing some subtasks related to bed–wheelchair transfer on admission. Therefore, identifying these non-independent subtasks and determining the extent of assistance needed early in hospitalization can help in developing a specific practice plan promptly. Special attention should be given to subtasks such as manipulating bed rails and wheelchairs, where the percentage of independent patients remained low even at the endpoint.

In contrast, patients in Cluster 2, who were not independent in many subtasks during early hospitalization but retained some motor function, have a high probability of becoming independent during prolonged hospitalization. Therefore, intervention strategies for many patients in this group should be designed with the goal of achieving independence on all subtasks. However, approximately one-third of the patients did not become independent in challenging subtasks, such as manipulating bed rails, a wheelchair, and pressing a nurse call button. A case study reported that an intervention combining errorless learning and spaced retrieval training was effective for a dementia patient who could not remember the procedures of pre-transfer wheelchair manipulation (47). The difficulty in remembering movement procedures due to cognitive decline is similar in patients with stroke, suggesting that this strategy may be effective for patients with stroke who are not independent in pre-transfer tasks. However, previous studies have shown that these subtasks related to transfer preparation are difficult to learn during hospitalization for patients who have not achieved independence on admission (23, 48). Therefore, if repeated practice does not lead to independence in these subtasks, continued practice until achieving independence may be less effective. In such cases, compensatory strategies such as environmental adjustments or the introduction of assistive devices (49, 50) are recommended rather than persisting with practice to achieve independence. Alternatively, patients with high ambulatory ability but difficulty in wheelchair manipulation may achieve early mobility independence by changing from a wheelchair to walking, which eliminates the need to master wheelchair manipulation. Patients with stroke often begin practising transfers using a wheelchair instead of walking first, as walking is more challenging than bed–wheelchair transfers for them (51–54). However, as approximately one-third of the patients in Cluster 2 no longer required bed-wheelchair transfers due to their walking independence, we believe that if walking independence is expected during hospitalization, it may be more efficient for patients to dedicate their time to practise aimed at improving mobility through walking rather than transferring at an early stage in the rehabilitation process. For patients who need transfer practice because they have not yet determined whether they will walk or use a wheelchair on discharge or because they need to reduce the amount of assistance they need for transfers during their daily life in the hospital, practising main tasks, such as standing and changing direction in a standing position, would be more effective than preparatory tasks, such as wheelchair manipulation, which results in a relatively low chance of independence.

Patients in Cluster 3, requiring assistance with many subtasks on admission and experiencing severe motor dysfunction, had a high probability of requiring assistance with many subtasks during hospitalization. Therefore, the focus of practice should be on subtasks that have the potential to reduce required assistance. At least two-thirds of patients will eventually need assistance with certain subtasks, such as wearing shoes, manipulating bed rails/wheelchairs, and getting up and lying down. When patients do not show substantial performance improvements in these subtasks after a certain amount of initial practice, practising subtasks that have a high probability of being performed with supervision/verbal assistance or independently, e.g., “Keep sitting at the bedside”, “Unlock the wheelchair brakes”, and “Manoeuvre the wheelchair”, should be prioritized. In addition, it may be necessary to develop strategies according to the specific needs of the patient and their family, as both the patient and their family prioritize independence during transfer (55), and minimizing the caregiver’s burden is a goal of the transfer. For example, practising main tasks may be prioritized to reduce the physical care burden on the family members caring for the patient.

### Limitations

There are several limitations to this study. First, because this was a single-centre study, the results may be influenced by facility-specific conditions, such as criteria for determining patient independence and the hospital room environment. In addition, because the target population was limited to patients admitted to the rehabilitation hospital, changes in transferring ability during the acute phase and after discharge remain unclear. Consequently, the present results may not fully represent the overall time-course of patients’ transferring ability. Therefore, future studies should investigate changes in transfer ability during the acute and chronic phases of patients after stroke in a multicentre setting to identify specific changes occurring in each phase. Understanding a series of processes for acquiring transfer skills from the acute to the chronic phase after stroke would provide valuable insight for developing specific practice strategies for each phase. Further, the influence of patient characteristics (e.g., spasticity and unilateral spatial neglect) on outcomes was not clarified in the present study. In addition, the time-course of acquiring transfer skills during hospitalization (i.e., determining the cluster to which the patient belongs) cannot be determined from their status on admission, which would enable more tailored interventions for individual patients. These important topics can be addressed in future studies. Some may argue that the number of participants was limited in the present study. However, we believe that this is not the case. Forman’s criterion (56), one of the well-known calculations for sample size in cluster analysis, recommends that the number of cases should be five times the number of variables used for clustering (i.e., 25 variables × 5 = 125 patients). Therefore, the number of participants was sufficient to perform the cluster analysis.

### Conclusion

The degree of independence in bed-to-wheelchair transfers and their changing processes were classified into three different time-courses, each of which may require a different intervention strategy. The findings of this study can be used to develop tailored intervention strategies for each patient’s time-course of transfer independence in the early stages of hospitalization.

## Supplementary Material

TIME-COURSE FOR ACQUIRING TRANSFER INDEPENDENCE IN PATIENTS WITH SUBACUTE STROKE: A PROSPECTIVE COHORT STUDY

TIME-COURSE FOR ACQUIRING TRANSFER INDEPENDENCE IN PATIENTS WITH SUBACUTE STROKE: A PROSPECTIVE COHORT STUDY
