# Supplementary material for: TIME-COURSE FOR ACQUIRING TRANSFER INDEPENDENCE IN PATIENTS WITH SUBACUTE STROKE: A PROSPECTIVE COHORT STUDY
Source: J Rehabil Med. 2024 Oct 9;56:40055. doi: 10.2340/jrm.v56.40055 (PMC11481307; doi:10.2340/jrm.v56.40055)
Supplement: TIME-COURSE FOR ACQUIRING TRANSFER INDEPENDENCE IN PATIENTS WITH SUBACUTE STROKE: A PROSPECTIVE COHORT STUDY [file JRM-56-40055-s1.pdf]

Supplementary material has been published as submitted. It has not been copyedited, or typeset by Journal of Rehabilitation Medicine

Fig. S1. Bed–Wheelchair Transfer Tasks Assessment Form (BTAF)

General comments:

Patient name: Assessor: Date: ～ Time of the day:

Setting: ☐No consideration ☐Handrail ☐Transfer board

Score: 3, independent; 2, requires supervision or verbal assistance; 1, requires assistance; N, not applicable

		Task	Score	Comments
Bed to the wheelchair	Preparation	Press the nurse call button		
	On the bed	Take off the comforter		
		Manipulate the handrail for the bed		
		Roll over		
		Get up		
		Keep sitting on the bedside		
	Shoes	Wear shoes/brace		
	Preparation for transfer	Ready the wheelchair for transfer (the position of the wheelchair, brakes, and footrests)		
	Transfer	Stand up from the bed		
		Turn while standing		
		Sit on the wheelchair seat		
	Preparation for driving	Put the foot on the footrest		
		Unlock the wheelchair brakes		
	Move	Maneuver the wheelchair		
Wheelchair to the bed	Preparation	Press the nurse call button		
		Maneuver the wheelchair toward the appropriate place for transfer to the bed		
		Lock the wheelchair brakes		
		Take the foot off the footrest and place it on the ground		
	Transfer	Stand up from sitting in the wheelchair		
		Turn while standing		
		Sit on the bed		
		Keep sitting on the bedside		
	Shoes	Take off shoes/brace		
	Movement on the bed	Lie down on the bed		
		Put on the comforter		
