# Supplementary material for: TIME-COURSE FOR ACQUIRING TRANSFER INDEPENDENCE IN PATIENTS WITH SUBACUTE STROKE: A PROSPECTIVE COHORT STUDY
Source: J Rehabil Med. 2024 Oct 9;56:40055. doi: 10.2340/jrm.v56.40055 (PMC11481307; doi:10.2340/jrm.v56.40055)
Supplement: TIME-COURSE FOR ACQUIRING TRANSFER INDEPENDENCE IN PATIENTS WITH SUBACUTE STROKE: A PROSPECTIVE COHORT STUDY [file JRM-56-40055-s2.pdf]

Supplementary material has been published as submitted. It has not been copyedited, or typeset by Journal of Rehabilitation Medicine

Table SI. STROBE Statement—checklist of items that should be included in reports of observational studies

	Item No.	Recommendation	Page No.	Relevant text from manuscript
**Title and abstract**	1	(*a*) Indicate the study's design with a commonly used term in the title or the abstract	1	Time-course for acquiring transfer independence in patients with subacute stroke: a prospective cohort study
		(*b*) Provide in the abstract an informative and balanced summary of what was done and what was found	3	Objective: To clarify the time-course of longitudinal changes in the independence level of subtasks composing bed-wheelchair transfer among patients with stroke.<br>Design: Single-institution prospective cohort study.<br>Patients: One hundred thirty-seven consecutive post-stroke patients using wheelchair upon admission to the subacute rehabilitation wards.<br>Methods: The independence degree in each of the 25 transfer-related subtasks was assessed using the Bed-Wheelchair Transfer Tasks Assessment Form on a three-level scale every 2 weeks, from admission to the endpoint (either discharge or when achieving independent transfer). Patients were classified based on admission and endpoint assessment form scores using two-step cluster analysis.<br>Results: Patients were classified into three clusters. The first cluster included 50 patients who exhibited a greater independence level in all subtasks upon admission (52.0–100% of patients performed each subtask independently) and at the endpoint (64.0–100%). The second included 30 patients who showed less independence upon admission (0–27.8%) but achieved greater independence levels at the endpoint (44.4–97.2%). The third included 51 patients whose independence level remained low in many subtasks from admission (0–5.8%) until the endpoint (0–29.4%).<br>Conclusion: The independence level and its changing process during transfer were categorised into three time-courses, each requiring different intervention strategies.
**Introduction**				

Background/rationale	2	Explain the scientific background and rationale for the investigation being reported	5, 6	Patients with stroke exhibit diverse clinical characteristics influenced by the location or volume of the brain damage (24–26) and demographic characteristics, such as age and sex. Therefore, the processes towards independence may also show different time-courses due to the interaction between the difficulty of each element of the transfer skill (23) and individual clinical and demographic characteristics. If so, tailored rehabilitation strategies are essential, especially for groups of patients with different time-courses in gaining transfer skills independence. Patients anticipating difficulty in acquiring the required independence level may prioritise simpler subtasks and utilise compensatory measures, such as assistive devices, for difficult-to-acquire subtasks. Understanding potential processes toward independence in transfer-related subtasks and identifying specific subtasks that tend to remain dependent can help patients prioritise which subtasks to practice and develop effective strategies for promoting independence.
Objectives	3	State specific objectives, including any prespecified hypotheses	5	This prospective cohort study aimed to clarify the differences in time-course and associated factors toward independence in wheelchair-to-bed transfer-related subtasks among patients with stroke. We classified patients into subtypes based on the time-course of changes in the independence level of subtasks during hospitalisation and investigated the characteristics of their time-course and patients' demographics within each subtype.
**Methods**				
Study design	4	Present key elements of study design early in the paper	6	This single-centre prospective cohort study adhered to the Strengthening the Reporting of Observational Studies in Epidemiology Statement guidelines (Supplementary table) (27).
Setting	5	Describe the setting, locations, and relevant dates, including periods of recruitment, exposure, follow-up, and data collection	6	The study was conducted in the Kaifukuki Rehabilitation Ward of Tokyo Bay Rehabilitation Hospital, a 160-bed facility in Japan. This ward provides subacute intensive rehabilitation covered by Japan's medical insurance system, admitting patients with stroke within 2 months of symptom onset for a maximum stay of 6 months (28). The sample size was determined based on the planned study period (April 2016 and March 2017).

Participants	6	(*a*) *Cohort study*—Give the eligibility criteria, and the sources and methods of selection of participants. Describe methods of follow-up<br>*Case-control study*—Give the eligibility criteria, and the sources and methods of case ascertainment and control selection. Give the rationale for the choice of cases and controls	6	Patients hospitalised after stroke were consecutively recruited between April 2016 and March 2017. Inclusion criteria comprised first ischemic or haemorrhagic stroke, hemiparesis with apparent unilateral motor paresis on the motor items in the Stroke Impairment Assessment Set (SIAS) upon admission, use of wheelchair upon admission for daily mobility, and provision of consent via a legal representative.
		Cross-sectional study—Give the eligibility criteria, and the sources and methods of selection of participants	7	Assessments were conducted every 2 weeks from admission until reaching one of the following endpoints: 1) Independence, when all subtasks were rated as "3, independent" or "N, not applicable," or when the patient received permission from the medical team to perform the transfer alone even if some subtasks were rated "2, requiring supervision or verbal assistance" or "1, requiring physical assistance" (i.e., if a patient was judged to be independent based on an assessment by the medical team separately from the study, we defined the previous 2-week assessment time as the endpoint, even if not all subtasks were rated as "3"); 2) Mobility change, when patients no longer used a wheelchair because they began to ambulate; 3) Discharge, when participants were discharged from the hospital regardless of the independence level.
		(*b*) *Cohort study*—For matched studies, give matching criteria and number of exposed and unexposed<br>*Case-control study*—For matched studies, give matching criteria and the number of controls per case	NA	NA
Variables	7	Clearly define all outcomes, exposures, predictors, potential confounders, and effect modifiers. Give diagnostic criteria, if applicable	6, 7	BTAF is a tool developed specifically to assess the bed-to-wheelchair transfer of patients with paretic stroke (Supplementary figure). The BTAF classifies a series of transfer tasks into 25 subtasks (Table 1). Each subtask independence level is assessed on a three-point scale: 3 for independent (participant can complete the task by themselves without any assistance by a therapist); 2, requiring supervision or verbal assistance (participant can complete a task under supervision or verbal assistance by a therapist); 1, requiring physical assistance (participant needs physical assistance from a therapist, such as locking the wheelchair brakes and manoeuvring the wheelchair to the appropriate position) to complete the task; and N, not applicable (participant

				does not need to perform the task: for example, the task of "put the foot on the footrest" can be applied only to those using wheelchairs with footrests). The mean subtask score was calculated to indicate the overall BTAF score for each patient. Subtasks marked as "N, not applicable" were excluded, and the mean score was calculated from the remaining subtasks (i.e. those judged as "1", "2" or "3"). For example, if 2 of the 25 subtasks were marked as "N", the mean score was calculated based on the other 23 subtasks. This assessment was demonstrated to have good reliability and validity (22).<br><br>Referring to Table 1
Data sources/ measurement	8*	For each variable of interest, give sources of data and details of methods of assessment (measurement). Describe comparability of assessment methods if there is more than one group	6	The patients' occupational therapist assessed the independence level of bed-wheelchair transfer by observing the actual performance in the hospital room using the BTAF (22).
			7	During the assessment, patients were instructed to perform bed-to-wheelchair transfer and wheelchair-to-bed transfers daily for 3 days. The lowest score of the three was used as the one representing the independence level of the subtask (if a patient's performance for one subtask was scored as "3", "3", and "2","2" was adopted).
Bias	9	Describe any efforts to address potential sources of bias	7	The BTAF assessment was conducted by well-trained occupational therapists with daily clinical practice in the hospital.
Study size	10	Explain how the study size was arrived at	6	The sample size was determined based on the planned study period (April 2016 and March 2017).

Continued on next page

Quantitative variables	11	Explain how quantitative variables were handled in the analyses. If applicable, describe which groupings were chosen and why	8	In the cluster analysis, the assessment results at two time points were used: at admission and at the endpoint. These were combined into categorical variables (e.g. "1–2" for a patient rated "1" upon admission and "2" at the endpoint), and 25 categorical variables per patient were evaluated.
			8	To characterise clusters, we calculated the percentage of patients corresponding to each of the BTAF scales (3, 2, 1, N) for each subtask at admission and at the endpoint for each cluster.
Statistical methods	12	(*a*) Describe all statistical methods, including those used to control for confounding	7, 8	We adopted a two-step cluster analysis to classify patients into subgroups based on the time-course of independence in bed-wheelchair transfer subtasks.
			8	The two-step cluster analysis was employed because it can include categorical variables, and the number of best-fitting clusters can be automatically determined. Log-likelihood was used as the distance measure, and Schwarz's Bayesian criterion was used to determine the cluster number. Subsequently, clustering quality was evaluated using silhouette coefficients. The coefficients range from -1 to 1, with -1 to 0.2 indicating poor; 0.2 to 0.5, fair; and ≥ 0.5, good (39).
			8	Demographic data were compared among the clusters to identify patient characteristics, using the Chi-square test for nominal data, one-way analysis of variance for proportional scale data, and the Kruskal–Wallis test for ordinal data.
			8	Cluster analysis and the subsequent analyses were performed using SPSS version 28 (IBM Corp., Armonk, NY, USA). Any p-values < 0.05 were considered statistically significant.
		(*b*) Describe any methods used to examine subgroups and interactions		NA
		(*c*) Explain how missing data were addressed		NA

		(*d*) *Cohort study*—If applicable, explain how loss to follow-up was addressed<br>*Case-control study*—If applicable, explain how matching of cases and controls was addressed<br>*Cross-sectional study*—If applicable, describe analytical methods taking account of sampling strategy		NA
		(*e*) Describe any sensitivity analyses		NA
**Results**				
Participants	13*	(a) Report numbers of individuals at each stage of study—eg numbers potentially eligible, examined for eligibility, confirmed eligible, included in the study, completing follow-up, and analysed	8	Among the 298 patients admitted with their first stroke during the study period, 137 consecutive patients who met the criteria were included (Figure 1).<br><br>Referring to Figure 1
		(b) Give reasons for non-participation at each stage		Referring to Figure 1
		(c) Consider use of a flow diagram		Referring to Figure 1
Descriptive data	14*	(a) Give characteristics of study participants (eg demographic, clinical, social) and information on exposures and potential confounders		Referring to Table 2
		(b) Indicate number of participants with missing data for each variable of interest		NA
		(c) *Cohort study*—Summarise follow-up time (eg, average and total amount)	7	The dataset of a single patient consisted of the independence ratings of the 25 BTAF subtasks for the number of times the assessment (mean 5.5 times) was completed.<br><br>Referring to Table 2
Outcome data	15*	*Cohort study*—Report numbers of outcome events or summary measures over time		Referring to Figure 2
		Case-control study—Report numbers in each exposure category, or summary measures of exposure		NA
		Cross-sectional study—Report numbers of outcome events or summary measures		NA

Main results	16	(*a*) Give unadjusted estimates and, if applicable, confounder-adjusted estimates and their precision (eg, 95% confidence interval). Make clear which confounders were adjusted for and why they were included	8	The patients were classified into three clusters using the two-step cluster analysis: 50 patients (36.5%) were assigned to Cluster 1, 36 (26.3%) to Cluster 2, and 51 (37.2%) to Cluster 3. Silhouette coefficients were 0.4, indicating that the clustering quality was "fair". Figure 2 shows the results of BTAF assessments for each subtask at admission and the endpoint in each cluster. Cluster 1 showed the highest percentage of "3" ratings for all items at admission (52.0–100%, mean 77.0%) and at the endpoint (64.0–100%, mean 88.5%). Cluster 2 displayed the highest percentage of "1" in all items at admission (44.4–88.8%, mean 63.0%) and the highest percentage of "3" at the endpoint (44.4–97.2%, mean 80.4%). Cluster 3 showed the highest percentage of "1" at admission (72.5–98.0%, mean 90.0%) and at the endpoint (31.3–84.3%, mean 57.8%) for most items (23/25, 92.0%). The time-course of mean BTAF subtask scores for individual patients is illustrated in Figure 3. Those in Cluster 1 had relatively high scores upon admission and showed rapid improvement. Those in Cluster 2 had low scores upon admission but significantly improved by the endpoint, while Cluster 3 participants, despite gradual improvement, maintained relatively low scores even at the endpoint.<br><br>Referring to Table 2
		(*b*) Report category boundaries when continuous variables were categorized		NA
		(*c*) If relevant, consider translating estimates of relative risk into absolute risk for a meaningful time period		NA

Continued on next page

Other analyses	17	Report other analyses done—eg analyses of subgroups and interactions, and sensitivity analyses	9	Table 2 presents the patient characteristics and results of statistical comparisons among the clusters. Statistically significant differences were observed in age (*F* [2,134] = 13.00, $p < 0.001$), sex (c^2 [2] = 8.62, $p = 0.013$), duration after stroke onset (*F* [2, 134] = 5.78, $p = 0.004$), duration of transfer assessment (*F* [2, 134] = 14.57, $p < 0.001$), MMSE (*H* [2] = 41.09, $p < 0.001$), FIM (motor: *H* [2] = 80.26, $p < 0.001$, cognitive: *H* [2] = 50.27, $p < 0.001$, and total scores: *H* [2] = 80.47, $p < 0.001$), and SIAS (upper extremity motor: *H* [2] = 24.96–34.25, $p < 0.001$, lower extremity motor: *H* [2] = 25.87–39.34, $p < 0.001$, and speech items: *H* [2] = 7.02, $p = 0.030$). A trend towards older age, longer post-onset and transfer assessment periods, fewer males, and lower cognitive and motor functions were evident from Cluster 1 to Cluster 3.<br>Regarding the reasons for ending the assessment (endpoint status), “independence of transferring” was the main reason for patients in Cluster 1 (68.0%), decreasing to 38.9% in Cluster 2 and 0% in Cluster 3. Simultaneously, the percentage of patients who ended the assessment due to “discharge” increased from Cluster 1 (6.0%), to Cluster 2 (27.8%), to Cluster 3 (92.2%, c^2 [4] = 86.52 $p < 0.001$). Notably, “independence of transferring with changing mobility from wheelchair to walking” was the most common reason in Cluster 2 (33.3%).<br>Regarding the independence level of each subtask for each cluster (Figure 2), the subtasks with the lowest percentage of “3” at the endpoint were those related to the preparation for transfer, such as preparing bed rails, nurse calls, and wheelchairs in Cluster 1 and Cluster 2. In particular, Cluster 2 contained at least one-third of dependent participants (i.e. who were rated as “2” or “3”) in subtasks such as “Press the nurse call button (bed to wheelchair)”, “Manipulate the bed handrail”, “Wear shoes/brace”, “Ready the wheelchair for transfer”, “Press the nurse call button (wheelchair to bed)”, and “Take the foot off the footrest”. Conversely, more than 90% of patients were rated “3” in the subtasks of “Roll over”, “Keep sitting on the bedside (bed to wheelchair)”, “Stand up from the bed”, “Sit on the wheelchair seat”, “Maneuver the wheelchair”, “Stand up from sitting in the wheelchair”, “Sit on the bed”, and “Keep sitting on the bedside (wheelchair to bed)”. In Cluster 3, only two subtasks, “Keep sitting on

				the bedside (bed to wheelchair)" and "Keep sitting on the bedside (wheelchair to bed)", had the highest percentage of "2" at the endpoint, while the other subtasks had the highest percentage of "1" both upon admission and at the endpoint. Especially in subtasks including "Manipulate the handrail for the bed", "Wear shoes/brace", "Ready the wheelchair for transfer", "Manoeuvre the wheelchair to the bed", "Take the foot off the footrest", and "Take off shoes/brace", at least two-thirds of the patients remained classified as "1" at the endpoint (indicating a need for assistance). In contrast, in the subtasks "Press the nurse call button (bed to wheelchair)", "Keep sitting at the bedside (bed to wheelchair)", "Unlock the wheelchair brakes", "Manoeuvre the wheelchair", "Press the nurse call button (wheelchair to bed)", and "Keep sitting at the bedside (wheelchair to bed);" more than half the patients were rated as "3" or "2" in endpoint, and relatively few patients remained rated as "1".
**Discussion**				
Key results	18	Summarise key results with reference to study objectives	10	To understand the time-course toward independence in transfer-related subtasks upon admission, we classified patients with subacute stroke based on the time-course in the level of independence of subtasks comprising bed-wheelchair transfer into three clusters: Cluster 1, patients who showed near independence in many subtasks upon admission and then became independent early during hospitalisation; Cluster 2, patients who required assistance upon admission but became independent during hospitalisation; Cluster 3, patients requiring assistance upon admission and remaining dependent at discharge.
Limitations	19	Discuss limitations of the study, taking into account sources of potential bias or imprecision. Discuss both direction and magnitude of any potential bias	12	There are several limitations to this study. First, because this was a single-centre study, the results may be influenced by facility-specific conditions, such as criteria for determining patient independence and the hospital room environment. In addition, since the target population was limited to patients admitted to the rehabilitation hospital, changes in transferring ability during the acute phase and after discharge remain unclear. Consequently, the present results may not fully represent the overall time-course of patients' transferring ability. Therefore, future studies should investigate changes in transfer ability during the acute and chronic phases of patients after stroke in a multicentre

				setting to identify specific changes occurring in each phase. Understanding a series of processes for acquiring transfer skills from the acute to the chronic phase after stroke would provide valuable insight for developing specific practice strategies for each phase. Further, the influence of patient characteristics (e.g., spasticity and unilateral spatial neglect) on outcomes was not clarified in the present study. In addition, the time-course of acquiring transfer skills during hospitalisation (i.e. determining the cluster to which the patient belong) cannot be determined from their status upon admission, which would enable more tailored interventions from individual patients. These important topics can be addressed in future studies. Some may argue that the number of participants were limited in the present study. However, we believe that this is not the case. Forman's criterion (56), one of the well-known calculations for sample size in cluster analysis, recommends that the number of cases should be five times the number of variables used for clustering (i.e., 25 variables × 5 = 125 patients). Therefore, the number of participants was sufficient to perform the cluster analysis.
Interpretation	20	Give a cautious overall interpretation of results considering objectives, limitations, multiplicity of analyses, results from similar studies, and other relevant evidence	10	The study identified three types of patients after stroke based on their time-course of independence in the subtasks comprising transfer. These findings corroborate a previous study on the time-course of FIM composite scores in patients with stroke admitted to a subacute rehabilitation unit (40). The previous study identified three types of patients: those with high scores upon admission and at discharge, those with intermediate scores upon admission that improved significantly by their discharge, and those with low scores throughout their hospital stay (40). Importantly, the existence of patients who required assistance upon admission but whose level of independence improved significantly during hospitalisation has been widely observed in many studies that examined the time-course of independence levels in ADL after stroke (41–44).<br>Comparing patient characteristics across clusters, we observed that younger age, shorter post-onset time, and duration between the initial and endpoint assessments were associated with higher independence percentages, progressing from Cluster 3 to 1. Additionally, male patients with higher

				cognitive and motor functions were more prevalent in clusters with greater independence. Previous studies have consistently reported a similar association, linking younger age (9–12), male sex (11, 13–15), shorter post-onset time (11, 45), and mild motor (13, 16), cognitive (14, 16), and language (46) dysfunctions to improved ADL in patients with stroke. The present findings indicate that many patient characteristics showed gradual changes from Cluster 1 to Cluster 3, suggesting that multiple characteristics, such as the severity of poststroke sequelae and other factors associated with the level of ADL independence (e.g. age and sex) (9–16, 45), rather than a single characteristic, contribute to classifying the patient groups.
Generalisability	21	Discuss the generalisability (external validity) of the study results	12	First, because this was a single-centre study, the results may be influenced by facility-specific conditions, such as criteria for determining patient independence and the hospital room environment. In addition, since the target population was limited to patients admitted to the rehabilitation hospital, changes in transferring ability during the acute phase and after discharge remain unclear. Consequently, the present results may not fully represent the overall time-course of patients' transferring ability. Therefore, future studies should investigate changes in transfer ability during the acute and chronic phases of patients after stroke in a multicentre setting to identify specific changes occurring in each phase. Understanding a series of processes for acquiring transfer skills from the acute to the chronic phase after stroke would provide valuable insight for developing specific practice strategies for each phase.
**Other information**				
Funding	22	Give the source of funding and the role of the funders for the present study and, if applicable, for the original study on which the present article is based		NA

*Give information separately for cases and controls in case-control studies and, if applicable, for exposed and unexposed groups in cohort and cross-sectional studies.

Note: An Explanation and Elaboration article discusses each checklist item and gives methodological background and published examples of transparent reporting. The STROBE checklist is best used in conjunction with this article (freely available on the Web sites of PLoS Medicine at http://www.plosmedicine.org/, Annals of Internal Medicine at http://www.annals.org/, and Epidemiology at http://www.epidem.com/). Information on the STROBE Initiative is available at www.strobe-statement.org.
